# Resilience in Elders of the Sardinian Blue Zone: An Explorative Study

**DOI:** 10.3390/bs8030030

**Published:** 2018-02-26

**Authors:** Maria Chiara Fastame, Paul Kenneth Hitchcott, Ilaria Mulas, Marilena Ruiu, Maria Pietronilla Penna

**Affiliations:** Department of Pedagogy, Psychology, Philosophy, University of Cagliari, Via Is Mirrionis 1, 09123 Cagliari, Italy; paul.hitchcott@unica.it (P.K.H.); imulas89@gmail.com (I.M.); marilenaruiu@gmail.com (M.R.); penna@unica.it (M.P.P.)

**Keywords:** aging, resilience, psychological well-being, depression, blue zone

## Abstract

**Background**: older adults from the Sardinian Blue Zone self-report low depressive symptoms and high psychological well-being. However, the role of dispositional resilience as a determinant of these characteristics is unknown. **Objectives**: the current study had three aims. First, to investigate associations among several putative predictors, including dispositional resilience and three established markers of positive and negative mental health. Second, to determine if gender differences in dispositional resilience, independent of age and cognitive impairment, are present in this population. Third, to examine the relative importance of the predictors of self-reported mental health and well-being. **Methods**: 160 elders were recruited in the Sardinian Blue Zone. The participants completed self-report measures of dispositional resilience, satisfaction with social ties, physical health, depressive symptoms, and psychological well-being. **Results**: trait resilience was significantly associated with predictors and markers of mental health. Males had significantly greater trait resilience. In regression analyses, dispositional resilience and satisfaction with social ties were significant predictors of all markers of mental health. Other factors were significantly associated only with certain markers. **Conclusions**: trait resilience and strong social ties appear to be key determinants of the high mental health of Sardinian Blue Zone older adults.

## 1. Introduction

Although many physical and psychological abilities decline during late adulthood, considerable individual differences have been observed. A subset of older adults shows superior functioning across multiple domains, such as physical and cognitive health, a state termed ‘successful ageing’ [1]. Although there is no universally accepted definition or metric of successful ageing, there is growing acceptance of the importance of its psychological components. The most commonly studied of these are perceived well-being and various facets of mental health. Low levels of depressed mood, motivation and morale, and, conversely, high levels of life satisfaction, autonomy, and purpose in life are considered important indicators of a person’s ability to cope with the inevitable challenges and losses that are associated with later life. An extensive literature showing that these characteristics are associated with a pattern of superior physical, psychological, social functioning, and even longevity [2,3,4], underscores this view.

### 1.1. Successful Aging: A Resilience Approach

The term ‘resilience’ has a variety of meanings in the literature, but, in broad terms, refers to positive adaptation in the face of adversity. In this sense, there is there is considerable correspondence between resilience and successful aging concepts. Early reviews [5] proposed that childhood resilience derives from three resource categories: individual (e.g., personality traits), interpersonal/social (e.g., family ties), and external (e.g., health and education services), and, more recently, models of successful ageing based on the same categories have been developed [6]. Further similarities also exist in terms of the approaches that have been identified as most likely to advance understanding. These include calls to assess the relative contribution of different resource categories in relation to positive as well as negative outcomes [6,7,8,9]. The present study was conducted concordant with this guidance to address limited understanding concerning the role of social ties and dispositional resilience in the distinctive profile of robust psychological health observed in older adults from the Sardinian Blue Zone.

### 1.2. Sardinian Blue Zone: Psychological Characteristics

Older adults from the Sardinian Blue Zone display an unusual combination of low mental ill-health and high perceived well-being e.g., [10,11,12], which is indicative of robust psychological adjustment [13,14,15] and suggestive of high resilience/successful ageing. This pattern is, to an extent, unexpected because of the low socioeconomic status (SES) of this region [16]. Research elsewhere shows that low SES is associated with worse mental health, well-being and increased mortality [17,18]. Thus, the superior psychological characteristics (and longevity) that are present in older Blue Zone Sardinians exist despite a context of reduced external resources (e.g., limited education and healthcare), which known to support adaptation to the challenges of aging. Moreover, it suggests an enrichment of individual and/or interpersonal resources promoting psychological health may be present in this population [6]. As such, these individuals may represent a valuable resource for advancing the understanding of the role of these protective influences in successful aging [19,20,21]. However, until relatively recently, there has been limited investigation of this subject.

### 1.3. Sardinian Blue Zone: Social Support

Previous research has implicated social support as a key external resource in moderating the impact of the age-related adversities on indices of successful aging [6,22], and there has been speculation that the tight-knit communities within the Sardinian Blue Zone provide older adults with enhanced social support contributing to their increased longevity [23]. Preliminary evidence suggests that satisfaction with social relationships with friends and family also contributes to their mental health and well-being [20]. Older Blue Zone Sardinians are also known to have increased engagement in socially-oriented leisure activities relative to a matched control sample from northern Italy. Moreover, this increased engagement in leisure activities was significantly related to mental health and perceived well-being [21,22]. Thus, although relatively few studies have been conducted, there is growing evidence that social support contributes to the advantageous psychological characteristics displayed by older adults from the Sardinian Blue Zone.

### 1.4. Sardinian Blue Zone: Dispositional Resilience

To date, there has been very little investigation of the internal resources, which may also contribute to the psychological health of Sardinian Blue Zone older adults [23]. An obvious candidate is dispositional resilience. Various scales are available and tap into personality trait linked to effective, active, and flexible, in coping with internal and external sources of stress. Individuals scoring low on such scales show poor adaptation to, and recover slowly from, stressors [24,25]. These findings extend to older adults in whom it has been shown that, in the face of both major and daily stressors, high resilience is associated with better well-being and affect [26]. Since these are the same indices of psychological health that are found to be enhanced in older adults from the Sardinian Blue Zone, there is empirical precedent for examining the role of this personality trait.

### 1.5. Aims and Hypotheses

The current study aims were to examine: (1) the associations among dispositional resilience (internal resource) and satisfaction about relationships with family members and friends (external resource) and self-reported indices of physical and mental health; (2) the possible gender differences in resilience (independent from variance in general cognitive efficiency); and, (3) the relative importance of resilience, satisfaction with relationships, perceived physical health, general cognitive efficiency, and various socio-demographic characteristics (i.e., age, marital status) as predictors of mental health and well-being.

In relation to these aims, it was hypothesized that: (1) there would be significant positive correlations between both dispositional resilience and relationship satisfaction with self-reported mental health and well-being; (2) higher levels of resilience were predicted for males. Although similar levels of ego resilience have been observed in male and female young adults [25], this prediction was assumed from previous reports that older Sardinian men report fewer depressive symptoms and greater perceived well-being e.g., [27,28,29]; and, (3) both social ties and dispositional resilience were predicted to have significant associations with psychological health. This prediction was based on prior research in this population e.g., [27] and broadly comparable findings elsewhere e.g., [30]. Since no prior study has addressed this question in older Blue Zone Sardinians, no predictions were stated regarding the relative importance of the internal and social resources.

## 2. Method

### Participants

A sample of 160 community-dwelling elders, 69 males and 91 females, aged 64–96 years (mean age = 78.3, SD = 7.13) were recruited in Talana and Urzulei, two villages located in the Sardinian Blue Zone [23]. All of the respondents were recruited by the third and fourth authors through personal contacts, ensuring that participants’ attention was drawn to their right to decline to participate or withdraw from the research. No incentive for participation was offered. Eight participants had adjusted MMSE scores below 20 and were excluded from further testing. A further seven individuals declined to participate in the study. This high rate of participation (96%) suggests a low risk of recruitment bias. Overall, the final sample was composed of 152 volunteers. 

To take part in the study, participants had to meet the following criteria: (1) to have been born and be resident in the above-mentioned villages; (2) to be a descendent of families living in the Sardinian Blue Zone for at least two previous generations; (3) to be living in private accommodation (i.e., non-institutionalized); and, (4) to be cognitively intact or have only mild cognitive decline. This was assessed using the Mini-Mental State Examination test (MMSE) [31] and participants scoring ≥24/30 were considered cognitively intact, whereas a score between 20 and 23 indicated mild cognitive decline. The first two criteria ensured that all of the participants were members of families that were native to the Sardinian Blue Zone with similar exposure to the distinctive sociocultural characteristics of this region. The third criterion excluded bias due to institutional status, a potential confound of the main outcomes of the present study [32]. The final criterion was included because of prior evidence that significant cognitive decline undermines the validity of self-report measures of mental health and well-being [33].

Table 1 summarizes the socio-demographic characteristics of the sample based on participant age. Participants were classified as old (64–74 years), very old (75–84 years), or oldest old (≥85 years). There was no significant gender imbalance in the sample (*χ*^2^ = 3.02, df = 1, *p* = 0.08). As expected for older adults from this region [34], most participants (78.7%) had less than nine years of formal education. When, as in previous studies [29,35], education was dichotomized as either low (i.e., 1–8 years) or high (>8 years), a significant difference within the sample was observed (*χ*^2^ = 54.4, df = 1, *p* < 0.0005). 

## 3. Materials

The following instruments were presented:

The Mini-Mental State Examination (MMSE) [31]. This is a paper and pencil test assessing general cognitive efficiency. It is composed of 20 items assessing short- and long-term memory, visuo-motor integration, spatio-temporal orientation, attention, and mental calculation processes. Scores were adjusted for age and education level, as previously recommended [36].

The preliminary interview by Fastame and Penna [37], was conducted to obtain fundamental socio-demographic characteristics (e.g., education, civil status, gender, age) and lifestyle (e.g., time spent for outdoor and indoor leisure activities, gardening) of the participants. 

The Ego Resiliency Scale Revised (ER89–R) [38]. This is a revised version of Kremen and Block’s [39] ego resiliency inventory assessing the ability to adjust one’s level of control in agreement with situational demands. Although the paramount requirement for the present study was to have a validated Italian translation of a well-established measure of dispositional resilience, at least among younger samples, this scale has been reported to have a binary factor structure [38]. These two factors have been identified as optimal regulation (OR), which taps behavioral and temperamental characteristics promoting effective management of negative emotionality (e.g., low anger and anxiety, reduced disengagement), and openness to life experiences (OL) which taps behavioral and temperamental characteristics conducive to positive emotionality (e.g., high joy and vigor, increased engagement). Prior evidence has stressed that importance of positive emotionality in positive adaptation to adversity in older adults [39]. Separate examination of the role of the OL and OR components of ego resilience may, therefore, permit a more fine-grained analysis of the role of ego resilience in the mental health and well-being of older adults.

The inventory is composed of 10 items, six assessing OR, four assessing OL. Participants were asked to self-rate the frequency of some daily life behaviors along a 7-point Likert scale from 1 (never) to 7 (always). Responses were totaled as appropriate to generate separate OR and OL scores. Since each subscale was derived from <10 items, internal consistency was computed using inter-item correlation coefficients [40]. The observed internal consistencies of both sub-scales were both in the optimal range (OR 0.35; OL 0.42) described for this index [40]. 

The Center for Epidemiological Studies of Depression Scale (CES-D) [41,42] is a self-report measure of depressive symptoms during the past week on a four-point Likert scale from 0, (never or rarely) to 3 (most days or every day). A score ≥16/60 indicates a risk of depressive illness. The Cronbach’s alpha in the current sample was 0.83. 

Perceived physical health was assessed, as previously [31], using a single item. The participants rated their physical health on an 11-point Likert scale ranging from worst (0) to best (10) possible. 

The personal satisfaction (i.e., PS-PWAQ) scale of the Psychological Well-Being and Aging Questionnaire by De Beni et al. [43]. This is a self-report measure validated for Italian elders assessing the degree of satisfaction with life past and present, plus the expectation of future satisfaction. It was devised and validated for use in older Italian adults. Responses on a 4-point Likert scale ranging from 1 (never) to 4 (often) are recorded. The PS-PWAQ subscale is composed of 11 items, therefore the maximum score for this measure is 44. In the current sample the internal consistency was 0.81 (Cronbach’s alpha). 

The Warwick-Edinburgh Mental Well-Being Scale (WEMWBS) [44,45]. This self-report inventory comprises 12 items assessing psychological well-being during the past two weeks along a 5-point Likert scale ranging from 1 (i.e., never) to 5 (i.e., always). A mean score of 43.8 (SD = 6.7) has previously been reported for Italians over 57 years of age. In the present study, scale internal consistency was 0.75 (Cronbach’s alpha).

Satisfaction about family relationships was assessed, as previously [34], using a 2-item scale. Participants responded on an 11-point Likert scale regarding satisfaction with family member relationships during the past week. Zero expressed least satisfaction, 10 denoted maximum satisfaction. The inter-item correlation index was 0.75.

Satisfaction about relationships with friends and neighbors was assessed, as previously [34], using a two-item scale. Participants responded on an 11-point Likert scale regarding satisfaction with friend and neighbor relationships during the past week. Zero expressed least satisfaction, 10 denoted maximum satisfaction. The mean score between the answers provided to the two items was computed. The inter-item correlation index was 0.24.

## 4. Procedure

All of the participants were interviewed individually in their own home. After providing written informed consent, each participant first completed the MMSE, followed by the preliminary interview by Fastame and Penna [37]. Next, the presentation order of the remaining tools was counterbalanced. As in previous studies, all of the measure items were read aloud by an investigator who recorded the verbal responses of participants on separate response sheets for each participant. This procedure was adopted to minimize fatigue, especially among the oldest participants. All of the measures were completed within 60 min, approximately. 

The study was conducted in accordance with the ethical standards of the institutional research committee and with the 1964 Helsinki declaration and its later amendments. All of the participants provided written informed consent to indicate their willingness to participate.

The data were analyzed using SPSS (version 22.0) for Windows (IBM, SPSS Statistics, New York, NY, USA). Preliminary analyses were conducted to examine the sample composition in terms of numbers of males and females and education. Associations between dispositional resilience, perceived physical health, depressive symptoms, and personal satisfaction indices were calculated with and without controlling for variance in general cognitive efficiency using the Pearson correlation coefficient. Partial correlations were performed after entering MMSE scores as the covariate. Gender differences in dispositional resilience were examined using a multivariate analysis of covariance in which age and general cognitive efficiency were entered as the covariates.

The relationship between the independent variables (age, marital status, general cognitive efficiency, perceived physical health, satisfaction with family and non-family social ties and dispositional resilience) and the dependent variables (depressive symptoms, psychological well-being, and personal satisfaction) was examined using stepwise multiple regression. This method identified significant predictors of each of the dependent variables, while simultaneously removing those unimportant variables from the final regression model. This approach was adopted because of the exploratory aspects of the present study.

## 5. Results

### 5.1. Aim 1: Associations Among Study Variables

Initially, the associations among dispositional resilience, relationship satisfaction with friends and family, perceived physical health, depressive symptoms (CES-D), mental well-being (WEMWBS), and personal satisfaction (PS subscale of the PWAQ) were explored. To this end, a series of Pearson product-moment correlation coefficients were computed with and without controlling for the effect of MMSE. 

The data of primary interest concern the relationships between the OR and OL components of ego resilience and the other study variables. Based on the criteria suggested by Cohen [46], small significant positive correlations were found between the OR score and perceived physical health and satisfaction about relationships with family members (both r = 0.17, *p* = 0.03). Similarly, a small, though negative, correlation was found between the OR and depressive symptoms scores (CES-D; r = −0.26, *p* = 0.002). Moderately large correlations involving OR were found with mental well-being (WEMWBS; r = 0.38, *p* < 0.0005) and personal satisfaction (PWAQ-PS; r = 0.35, *p* < 0.0005). Regarding the OL component of ego resilience, small significant correlations were found with depressive symptoms (CES-D; r = −0.17, *p* < 0.04), satisfaction about relationships with friends (r = 0.22, *p* < 0.007) and mental well-being (WEMWBS; r = 0.26, *p* = 0.001) indexes, respectively. The same pattern of results was found when partial correlation coefficients were calculated controlling for variance in cognitive status (MMSE score). Table 2 illustrates these outcomes.

### 5.2. Aim 2: Gender Differences in Ego Resiliency

Second, a multivariate analysis of covariance (MANCOVA) was conducted to investigate the impact of gender on the OR and OL indexes. The extent to which gender differences were independent from variance in cognitive efficiency and age was determined by entering MMSE score and age as covariates. The Multivariate tests indicated a significant main effect of gender (Wilks’ Lambda = 0.90, df = 2;147, *p* = 0.0001), while neither the effect of age (Wilks’ Lambda = 0.99, df =2;147, *p* = 0.66) or MMSE score (Wilks’ Lambda = 0.99, df =2;147, *p* = 0.90) was significant. The main effect of gender was significant on both dimensions of ego resilience: OR [F(1,148) = 7.88, *p* = 0.006, η²p = 0.05] and OL [F(1,148) = 11.9, *p* = 0.001, η²p = 0.07]. As expected, men scored significantly higher on both the OR (M = 34.3, SD = 3.8 vs. M = 32.3, SD = 4.6) and OL (M = 20.7, SD = 4.3 vs. M = 18.1, SD = 4.5) dimensions of ego resilience. Table 3 summarizes these findings. 

### 5.3. Aim 3: Relative Importance of the Predictors of Psychological Health

Finally, three separate stepwise regression analyses were conducted. Each examined age, marital status, age, general cognitive efficiency, perceived physical health, satisfaction about relationships with family and non-family members, and the OL and OR dimensions of resilience as predictors of one of the three self-report measures of mental health (CES-D, WEMWBS and PS-PWAQ), in turn. 

When depressive symptomatology (CES-D measure) was entered as the dependent variable, it was found that marital status (b = −0.32, t = −4.6, *p* = 0.0005), perceived physical health (b = −0.25, t = −3.5, *p* < 0.001), OR score (b = −0.20, t = −2.9, *p* = 0.004), age (b = 0.17, t = 2.4, *p* = 0.02), and satisfaction about relationships with family members (b = −0.15, t = −2.2, *p* = 0.03) predicted 32% of the variance in the CES-D measure (corrected R^2^ = 0.32, F(5, 147) = 15.2, *p* < 0.0005). 

When mental well-being (WEMWBS measure) was examined, the analysis indicated that OR (b = 0.34, t = 4.92, *p* < 0.0005), satisfaction about relationship with friends (b = 0.30, t = 4.34 *p* < 0.0005), and marital status (b = 0.22, t = 3.22, *p* = 0.002) were significant predictors, accounting for 29% of the variance in the WEMWBS scores (corrected R^2^ = 0.29, F(3, 149) = 21.4, *p* < 0.0005). 

Lastly, perceived physical health (b = 0.31, t = 4.4, *p* < 0.0005), OR (b = 0.22, t = 3.0, *p* = 0.003), satisfaction about relationships with friends (b = 0.22, t = 3.02, *p* = 0.002), age (b = 0.22, t = 3.11, *p* = 0.002), and satisfaction about relationships with family members (b = 0.18, t = 2.4, *p* = 0.02) were significant predictors of perceived well-being (PS-PWAQ) accounting for 32% of the variance in this outcome (R^2^ corrected = 0.32, F(5, 147) = 14.97, *p* < 0.0005). These results are illustrated in Table 4. 

## 6. Discussion

The present study builds on previous research highlighting that the longevous population inhabiting the Sardinian Blue Zone possess an advantageous combination of psychological characteristics in the form of increased well-being and reduced mental ill-health. These characteristics are consistent with an enhanced capacity to cope with the challenges that are associated with aging and are among the most extensively investigated psychological markers of successful aging. 

The primary aim was to extend the understanding about the role of internal and external resources in these outcomes, particularly with respect to the role of dispositional resilience. Since there has been no previous investigation of this nature, the present research provides several novel findings. The anticipated binary correlations between dispositional resilience and all outcome measures used were observed. In addition, older males reported higher levels of dispositional resilience. Lastly, evidence that dispositional resilience operates in combination with other known and suspected resources to support mental health and well-being was obtained. In the stepwise regressions, dispositional resilience and relationship satisfaction were significant predictors of two of the three mental health and well-being outcomes that were studied. 

### 6.1. Ego Resilience and Mental Health

The term resilience has many meanings within the literature. When treated as a personality trait (e.g., ego resilience), it is possible to account for individual differences in the capacity to cope with stress and adversity. People with higher trait resilience recover more rapidly from both laboratory and real-world stressors [24,25]. Moreover, among older adults, higher dispositional resilience is associated with increased resistance to, and faster recovery from, the impact of stressors [26,47]. The findings of the present study are consistent with this broader literature. Participants’ OR scores were a significant predictor of two of the outcomes examined, depressive symptoms (CES-D), and mental well-being (WEMWBS). By contrast, OR scores were not significantly correlated with the personal satisfaction scale of the PWAQ. One possible explanation for this pattern is that the PWAQ-PS reflects a predominantly cognitive appraisal of life satisfaction (past, present, and future), whereas the other outcomes are more related to participants’ emotional state in the past two weeks. However, why OL scores, an index of the capacity to promote positive emotionality [38], were not significantly correlated with any of the outcome measures is less clear. Indeed, this finding is surprising given prior research showing that positive emotions mediate the association between ego resilience and the adaptation to adversity in older adults [48].

### 6.2. Sex Differences in Ego Resilience

The identification of sex differences in ego resilience is intriguing given previous reports that, at least among younger adults, levels do not differ between males and females [25]. At present, the cause of this difference is unclear although the distinctive cultural environment of the Sardinian Blue Zone could play a role. Block and Kramen [39] argued that overcoming societal pressure to control impulse expression was crucial for the development of ego resilience in women, whereas, the development of prosocial behaviors curbing aggression was argued to be crucial in men. The traditional, tight-knit nature of communities within the Sardinian Blue Zone would seem more conducive to the development of ego resilience in males. This idea is consistent with broader evidence highlighting that the superior longevity and mental health associated with this region are more evident in men and may derive from sociocultural influences, at least in part [16,23,27,28]. However, genetic factors could also play a role. Like most personality traits, resilience has a strong genetic component [49] and sex differences in specific polymorphisms that are linked to trait resilience have been observed previously [50]. 

### 6.3. Social Ties

Quality of social relationships with family members or friends is repeatedly identified to be a key factor promoting successful adaptation to stress and adversity [7], and, in older adults, is considered as an important external resource for successful aging [6]. Older adults with more satisfying social ties to others show better outcomes in terms of both positive and negative aspects of psychological health. Examples include, a reduced cardiovascular reactivity to stress [51], better emotional well-being following widowhood [52], and higher perceived well-being [53]. Preliminary evidence also links satisfaction with friend and family relationships to the superior psychological health of older adults from the Sardinian Blue Zone [34]. The present findings are consistent with this evidence and add to growing indications that that the lifestyle and culture of this region promote close social ties between family and community members, which contribute to the increased longevity and psychological health of this population [23,27,34]. Thus, satisfaction with either or both family and friends was consistently a predictor of all three of the outcomes that are examined in the present study.

### 6.4. Limitations and Future Research

The dimensionality of the ego resilience scale has been debated and the factor structure adopted here was originally determined in a much younger sample [38]. Whether this factor structure is age-invariant is unclear, although the modest correlation observed between OR and OL scores is consistent with the independence of these dimensions. However, participants’ OL scores were not a significant predictor of any aspect of psychological health, a finding that seems inconsistent with prior evidence [48]. Complete or partial replication of the present study in a sample sufficiently large to investigate the dimensionality of ego resilience is one option. A comparison with different measures of dispositional resilience e.g., [54] would also be helpful. 

There are similar issues arising from the use of relatively simple measures of relationship satisfaction. These measures have previously been employed in the Sardinian Blue Zone population to gain preliminary understanding about why this population reports unusually high psychological well-being [34]. Although the present data are consonant with this previous research, deeper understanding of the role of social support and relationship satisfaction in the Sardinian Blue Zone is likely to require the use of more sophisticated instruments. This is an attractive avenue for future research given speculation and growing evidence concerning increased social connectedness within this community [23,27,34]. As highlighted above, one intriguing idea is that intrinsic social connectedness of Sardinian communities fosters development of trait resilience, particularly among males. Studies involving the measurement of ego resilience throughout adulthood could shed light on this possibility.

## 7. Conclusions

To summarize, the present study describes several new findings that extend understanding about the basis of the high mental health and well-being of older adults inhabiting the Sardinian Blue Zone. Moreover, the study answers the increasing calls for investigations of the relative contributions of internal and external resources on positive (e.g., perceived well-being), as well as negative (physical and mental ill-health) outcomes e.g., [8,47,55]. Thus, dispositional resilience and satisfaction with social ties emerged as key predictors of all the outcomes (positive or negative) in the present study. The data also suggest that the previously described psychological advantages of older Blue Zone men (higher mental health and well-being) is related to their having higher dispositional resilience.

## Figures and Tables

**Table 1:** Socio-demographic characteristics of the sample.

			Groups			
	Old		Very Old		Oldest-Old	
	(64–74 Years)		(75–84 Years)		(≥85 Years)	
N	53		73		26	
Gender						
Males	21		33		13	
Females	32		40		13	
Age (years)	M = 70.5 (2.7)		M = 79.4 (3)		M = 87.8 (2.4)	
Marital status						
Single/widowed	15		27		14	
Married/engaged	38		46		12	
Education (years)						
	Males	Females	Males	Females	Males	Females
0–8	11	21	26	37	12	12
>8	10	11	7	3	1	1

Socio-demographic characteristics by age group. M indicates mean score, standard deviation scores are illustrated in brackets.

**Table 2:** Pearson’s product moment coefficients with and without controlling for the effect of MMSE.

	OR (1)	OL (2)	PHYS (3)	FAMILY (4)	FRIENDS (5)	PS-PWAQ (6)	WEMWBS (7)	CES-D (8)
1	1	0.30 **	0.17 *	0.17 *	0.14	0.35 **	0.38 **	−0.26 **
2	0.24 *	1	−0.01	0.06	−0.10	0.11	0.26 **	−0.17 *
3	0.16 *	**−0.02**	1	0.13	0.12	0.36 **	0.20 *	−0.37 **
4	0.17 *	**0.06**	**0.13**	1	0.30 **	0.31 **	0.29 **	−0.25 **
5	**0.11**	0.17 *	**0.12**	0.31 **	1	0.30 **	0.38 **	−0.16
6	0.36 **	**0.11**	0.37 **	0.35 **	0.31 **	1	0.54 **	−0.46 **
7	0.38 **	0.26 *	**0.20**	0.29 **	0.37 **	0.55 **	1	−0.47 **
8	−0.26 **	−0.17 *	−0.37 **	−0.26 **	**−0.15**	−0.46 **	−0.47 **	1

* *p* < 0.05; ** *p* ≤ 0.001. Correlation Matrix among all study variables. Dispositional resilience optimal regulation (OR) and openness to life experiences (OL) dimensions, perceived physical health (PHYS), personal satisfaction about relationships with family members (FAMILY) or friends (FRIENDS), personal satisfaction index of the Psychological Well-Being and Aging Questionnaire (PS-PWAQ), the Warwick-Edinburgh Mental Well-Being Scale (WEMWBS) and self-reported depressive signs (CES-D). Partial correlations were computed controlling for the effect of MMSE score and are reported in bold.

**Table 3:** Summary of multivariate analysis of covariance (MANCOVA) analysis.

		df	SS	MS	F	*p*	η²p	M _males_	M _females_
OL	Main effect								
	Gender	1	238.19	238.19	11.9	0.001	0.07	20.7	18.1
	Covariates								
	Age	1	0.243	0.243	0.012	0.91			
	Cognitive efficiency	1	2.53	2.53	0.13	0.72			
OR	Main effect								
	Gender	1	147.5	147.5	7.88	0.006	0.05	34.3	32.3
	Covariates								
	Age	1	13.76	13.76	0.73	0.39			
	Cognitive efficiency	1	0.714	0.714	0.038	0.84			

Summary of MANCOVA analysis examining the effect of gender on the ego resilience dimensions of openness to life experience (i.e., OL) and optimal regulation (i.e., OR) scores controlling for the effect of age and general cognitive efficiency.

**Table 4:** Stepwise linear regression analyses illustrating the predictors of the general measure of perceived subjective well-being across the participants.

	**Depression**											
**Model**	**Unstandardized Coefficients**		**Standardized Coefficients**			**Correlations**			**Model Summary and Change Statistics**			
	**B**	**SE**	**β**	**t**	***p***	**Zero-Order**	**Partial**	**Part**	**R^2^**	**adj. R^2^**	**SE of Estimate**	**F Chg.**
1. Constant	20.76	1.24		16.71	<0.001				0.153	0.147	9.130	26.36 **
Marital status	–8.00	1.56	–0.39	–5.13	<0.001	–0.39	–0.39	–0.39				
Physical health			–0.32	–4.45	<0.001		–0.35					
Optimal regulation			–0.25	–3.41	0.001		–0.27					
Openness to life experiences			–0.12	–1.63	0.105		–0.13					
Age			0.19	2.45	0.16		0.2					
Satisfaction about			–0.23	–3.16	0.002		–0.25					
family ties												
Satisfaction about non-family ties			–0.10	–1.30	0.195		–0.11					
General cognitive efficiency			–0.05	–0.60	0.547		–0.50					
2. Constant	28.25	2.05		13.79	<0.001				0.255	0.244	8.594	19.81 **
Marital status	–7.17	1.48	–0.35	–4.85	<0.001	–0.39	–0.37	–0.35				
Physical health	–1.26	0.28	–0.32	–4.45	<0.001	–0.36	–0.35	–0.32				
Optimal regulation			–0.20	–2.87	0.005		–0.23					
Openness to life experiences			–0.13	–1.90	0.059		–0.16					
Age			0.15	2.03	0.044		0.17					
Satisfaction about family ties			–0.19	–2.75	0.007		–0.22					
Satisfaction about non-family ties			–0.07	–0.94	0.349		–0.08					
General cognitive efficiency			–0.06	–0.81	0.42		–0.67					
3. Constant	42.62	5.39		7.9	<0.001				0.295	0.280	8.387	8.23 *
Marital status	–7.18	1.44	–0.35	–4.97	<0.001	–0.39	–0.38	–0.35				
Physical health	–1.12	0.28	–0.29	–4.02	<0.001	–0.37	–0.32	–0.28				
Optimal regulation	–0.46	0.16	–0.20	–2.87	0.005	–0.26	–0.23	–0.20				
Openness to life experiences			–0.09	–1.25	0.213		–0.10					
Age			0.18	2.55	0.012		0.21					
Satisfaction about family ties			–0.17	–2.40	0.018		–0.20					
Satisfaction about non-family ties			–0.05	–0.71	0.48		–0.06					
General cognitive efficiency			–0.07	–0.98	0.328		–0.08					
4. Constant	22.67	9.44		2.4	0.018				0.326	0.307	8.231	6.52 *
Marital status	–6.62	1.43	–0.32	–4.62	<0.001	–0.39	–0.36	–0.32				
Physical health	–1.02	0.28	–0.26	–3.68	<0.001	–0.37	–0.29	–0.25				
Optimal regulation	–0.51	0.16	–0.23	–3.26	0.001	–0.26	–0.26	–0.22				
Openness to life experiences			–0.09	–1.32	0.19		–0.11					
Age	0.27	0.1	0.18	2.55	0.012	0.25	0.21	0.17				
Satisfaction about family ties			–0.15	–2.22	0.028		–0.18					
Satisfaction about non-family ties			–0.21	–0.29	0.768		–0.02					
General cognitive efficiency			–0.06	–0.89	0.377		–0.07					
5. Constant	31.61	10.14		3.12	0.002				0.348	0.325	8.120	4.92 *
Marital status	–6.55	1.41	–0.32	–4.63	<0.001	–0.39	–0.36	–0.31				
Physical health	–0.96	0.27	–0.25	–3.50	0.001	–0.37	–0.28	–0.24				
Optimal regulation	– 0.46	0.16	–0.20	–2.90	0.004	–0.26	–0.24	–0.20				
Openness to life experiences			–0.09	–1.28	0.204		–0.11					
Age	0.25	0.1	0.17	2.39	0.018	0.25	0.2	0.16				
Satisfaction about family ties	–1.09	0.49	–0.15	–2.22	0.028	–0.25	–0.18	–0.15				
Satisfaction about non-family ties			–0.23	–0.31	0.757		–0.03					
General cognitive efficiency			–0.08	–1.16	0.247		–0.10					
	**Psychological Well-Being**											
**Model**	**Unstandardized Coefficients**		**Standardized Coefficients**			**Correlations**			**Model Summary and Change Statistics**			
	**B**	**SE**	**β**	**t**	**p**	**Zero-Order**	**Partial**	**Part**	**R^2^**	**adj. R^2^**	**SE of Estimate**	**F Chg.**
1. Constant	23.45	4.33		5.42	<0.001				0.144	0.138	6.913	24.84 **
Marital status			0.27	3.67	<0.001		0.29					
Physical health			0.14	1.83	0.069		0.15					
Optimal regulation	0.64	0.13	0.38	4.98	<0.001	0.38	0.38	0.38				
Openness to life experiences			0.18	2.38	0.019		0.19					
Age			–0.10	–1.31	0.19		–0.19					
Satisfaction about family ties			0.22	2.96	0.004		0.24					
Satisfaction about non-family ties			0.34	4.71	<0.001		0.36					
General cognitive efficiency			0.48	0.63	0.531		0.05					
2. Constant	15.06	4.42		3.4	0.001				0.256	0.246	6.466	22.16 **
Marital status			0.22	3.22	0.002		0.26					
Physical health			0.11	1.47	0.143		0.12					
Optimal regulation	0.57	0.12	0.34	4.76	<0.001	0.38	0.36	0.34				
Openness to life experiences			0.14	1.85	0.066		0.15					
Age			–0.04	–0.53	0.598		–0.04					
Satisfaction about family ties			0.13	1.74	0.084		0.14					
Satisfaction about non-family ties	1.33	0.28	0.34	4.71	<0.001	0.38	0.36	0.33				
General cognitive efficiency			0.03	0.49	0.623		0.41					
3. Constant	13.81	4.31		3.21	0.002				0.305	0.291	6.268	10.40 *
Marital status	3.48	1.08	0.22	3.22	0.002	0.27	0.26	0.22				
Physical health			0.08	1.17	0.244		0.24					
Optimal regulation	0.58	0.12	0.34	4.92	<0.001	0.38	0.38	0.34				
Openness to life experiences			0.12	1.62	0.11		0.13					
Age			–0.01	0.11	0.911		–0.09					
Satisfaction about family ties			0.14	1.87	0.063		0.15					
Satisfaction about non-family ties	1.2	0.28	0.3	4.34	<0.001	0.38	0.34	0.3				
General cognitive efficiency			0.04	0.57	0.571		0.05					
	**Personal Satisfaction**											
**Model**	**Unstandardized Coefficients**		**Standardized Coefficients**			**Correlations**			**Model Summary and Change Statistics**			
	**B**	**SE**	**β**	**t**	**p**	**Zero-Order**	**Partial**	**Part**	**R^2^**	**adj. R^2^**	**SE of Estimate**	**F Chg.**
1. Constant	33.4	0.99		33.58	<0.001				0.133	0.128	4.460	22.64 **
Marital status			0.08	1.07	0.29		0.09					
Physical health	0.69	0.14	0.36	4.76	<0.001	0.36	0.36	0.36				
Optimal regulation			0.3	4.08	0.001		0.32					
Openness to life experiences			0.12	1.53	0.129		0.13					
Age			0.2	2.65	0.009		0.21					
Satisfaction about family ties			0.27	3.59	<0.001		0.28					
Satisfaction about non-family ties			0.26	3.47	0.001		0.28					
General cognitive efficiency			–0.02	–0.22	0.823		–0.02					
2. Constant	23.12	2.69		8.59	<0.001				0.222	0.211	4.240	16.62 **
Marital status			0.08	1.16	0.25		0.1					
Physical health	0.59	0.14	0.31	4.24	<0.001	0.36	0.33	0.31				
Optimal regulation	0.3	0.08	0.28	3.87	<0.001	0.35	0.32	0.3				
Openness to life experiences			0.45	0.59	0.553		0.05					
Age			0.16	2.13	0.03		0.18					
Satisfaction about family ties			0.22	3.6	<0.001		0.25					
Satisfaction about non-family ties			0.23	3.24	0.001		0.26					
General cognitive efficiency			–0.003	–0.04	0.978		–0.003					
3. Constant	19.58	2.83		6.92	<0.001				0.275	0.260	4.108	10.52 *
Marital status			0.06	0.78	0.435		0.06					
Physical health	0.55	0.14	0.29	4.02	<0.001	0.36	0.32	0.28				
Optimal regulation	0.3	0.08	0.28	3.87	<0.001	0.35	0.31	0.27				
Openness to life experiences			0.08	0.11	0.914		0.01					
Age			0.21	2.97	0.003		0.24					
Satisfaction about family ties			0.16	2.24	0.027		0.18					
Satisfaction about non-family ties	0.59	0.18	0.23	3.24	0.001	0.3	0.26	0.23				
General cognitive efficiency			–0.01	–0.18	0.859		–0.01					
4. Constant	7.74	4.84		1.6	0.112				0.317	0.298	4.001	8.85 *
Marital status			0.08	1.2	0.23		0.1					
Physical health	0.61	0.13	0.32	4.53	<0.001	0.36	0.35	0.31				
Optimal regulation	0.26	0.08	0.24	3.44	0.001	0.35	0.28	0.24				
Openness to life experiences			0.002	0.03	0.977		0.002					
Age	0.15	0.05	0.21	2.97	0.003	0.14	0.24	0.2				
Satisfaction about family ties			0.18	2.42	0.017		0.2					
Satisfaction about non-family ties	0.68	0.18	0.27	3.83	<0.001	0.3	0.3	0.26				
General cognitive efficiency			–0.005	–0.07	0.941		–0.01					
5. Constant	4.44	4.95		0.9	0.371				0.344	0.321	3.936	5.85 *
Marital status			0.09	1.31	0.192		0.11					
Physical health	0.58	0.13	0.31	4.39	<0.001	0.36	0.34	0.3				
Optimal regulation	0.24	0.08	0.21	3.1	0.002	0.35	0.25	0.21				
Openness to life experiences			0.01	0.9	0.932		0.01					
Age	0.16	0.05	0.22	3.11	0.002	0.14	0.25	0.21				
Satisfaction about family ties	0.58	0.24	0.18	2.42	0.017	0.31	0.2	0.16				
Satisfaction about non-family ties	0.56	0.18	0.22	3.02	0.003	0.3	0.24	0.2				
General cognitive efficiency			0.01	0.18	0.859		0.01					

Predictors of self-reported depression, psychological well-being and personal satisfaction measures using perceived physical health, age, marital status (single versus married/engaged), general cognitive efficiency, personal satisfaction about relationships with family members, personal satisfaction about relationships with non-family members, optimal regulation and openness to life experiences scores as independent variables.
